# Decisions to attend holiday gatherings during COVID-19 and engagement in key prevention strategies: United States, January 2021

**DOI:** 10.1017/S0950268822000115

**Published:** 2022-02-09

**Authors:** Mary A. Pomeroy, Edward R. Hoover, Brianna L. Dumas, Katrina S. Kennedy, Beth Wittry, Mark E. Laughlin, Diane M. Harris, Laura Gieraltowski, Merissa A. Yellman, Amanda G. Garcia-Williams, Katherine E. Marshall

**Affiliations:** 1 CDC COVID-19 Response, Centers for Disease Control and Prevention, Atlanta, Georgia, USA; 2 National Center for Emerging and Zoonotic Infectious Diseases, Centers for Disease Control and Prevention, Atlanta, Georgia, USA; 3 National Center for Environmental Health, Centers for Disease Control and Prevention, Atlanta, Georgia, USA; 4 National Center for Chronic Disease Prevention and Health Promotion, Centers for Disease Control and Prevention, Atlanta, Georgia, USA; 5 National Center for Injury Prevention and Control, Centers for Disease Control and Prevention, Atlanta, Georgia, USA

**Keywords:** COVID-19, SARS-COV-2, gatherings, food, prevention strategies, behaviors, mitigation measures, masking

## Abstract

Gatherings where people are eating and drinking can increase the risk of getting and spreading SARS-CoV-2 among people who are not fully vaccinated; prevention strategies like wearing masks and physical distancing continue to be important for some groups. We conducted an online survey to characterise fall/winter 2020–2021 holiday gatherings, decisions to attend and prevention strategies employed during and before gatherings. We determined associations between practicing prevention strategies, demographics and COVID-19 experience. Among 502 respondents, one-third attended in person holiday gatherings; 73% wore masks and 84% practiced physical distancing, but less did so always (29% and 23%, respectively). Younger adults were 44% more likely to attend gatherings than adults ≥35 years. Younger adults (adjusted prevalence ratio (aPR) 1.53, 95% CI 1.19–1.97), persons who did not experience COVID-19 themselves or have relatives/close friends experience severe COVID-19 (aPR 1.56, 95% CI 1.18–2.07), and non-Hispanic White persons (aPR 1.57, 95% CI 1.13–2.18) were more likely to not always wear masks in public during the 2 weeks before gatherings. Public health messaging emphasizing consistent application of COVID-19 prevention strategies is important to slow the spread of COVID-19.

## Introduction

Severe acute respiratory syndrome coronavirus 2 (SARS-CoV-2), the virus that causes Coronavirus Disease (COVID-19), is transmitted mainly through close contact with someone who is infected. By 1 July 2021, more than 33.6 million US COVID-19 cases and over 600 000 associated deaths had been reported [1]. Social gatherings, including religious gatherings, wedding receptions and family reunions, have been cited as sources of SARS-CoV-2 transmission [2–5] including breakthrough infections among those who are vaccinated [6]. During fall and winter months, holiday gatherings among friends and family often occur. Due to cooler temperatures, these gatherings may occur indoors, which increases the risk of SARS-CoV-2 transmission – especially when ventilation is poor [7,8]. These gatherings frequently centre around shared food and drinks, which mark time-honoured rituals that symbolise community, connection and tradition [9]. However, social gatherings that involve consuming food and drinks may contribute additional risk of SARS-CoV-2 transmission, since some key prevention strategies, such as wearing a mask, are not possible while actively eating and drinking.

The US Centers for Disease Control and Prevention (CDC) issued guidance on holiday gatherings from October to December 2020, which emphasised celebrating holidays only with persons who live in the same household, and also provided strategies for navigating holiday gatherings with non-household members (NHHMs) as safely as possible. The guidance stressed prevention strategies such as consistently wearing masks covering the nose and mouth, practicing physical distancing, improving ventilation, bringing one's own food and drink, and setting expectations with NHHMs about prevention strategies during and before gatherings.

From 25 November 2020 to 15 January 2021, cases of COVID-19 increased by 83% and deaths increased by 50% nationally [1]; gatherings around the holidays, especially those involving meals, may have contributed to this increase. However, there are limited data characterizing holiday gatherings during the 2020 holiday season and adherence to CDC's COVID-19 guidance. We characterised holiday gatherings involving meals with NHHMs by size, setting and engagement in prevention strategies; described factors influencing decisions to attend holiday gatherings with NHHMs; and identified characteristics and factors associated with engaging in prevention strategies during and before holiday gatherings.

## Methods

During 12–14 January 2021, 502 US adults aged ≥18 years completed an opt-in online survey administered by Porter Novelli Public Services and ENGINE Insights using the Lucid platform (31.5% response rate; 502/1594) [10,11]. The Lucid platform is comprised of multiple panel surveys. Survey respondents were selected through quota sampling, among a nationwide sample of survey volunteers from multiple panel surveys; individuals who had not taken a survey in the previous 20 waves of survey administration were eligible to participate. Responses were weighted by gender, age, community type, census region, race/ethnicity, household income, employment status and education to reflect US Current Population Survey proportions (Table 1) [12]. CDC licensed these data from Porter Novelli Public Services. While Porter Novelli Public Services and its vendors are not subject to CDC Institutional Review Board (IRB) review, they do adhere to all professional standards and codes of conduct set forth by the Council of American Survey Research Organizations (CASRO). Respondents are informed that their answers are being used for market research and they may refuse to answer any question at any time. No personal identifiers are included in the data file that is provided to CDC. Additionally, this activity was reviewed by CDC and was conducted consistent with applicable federal law and CDC policy.^1^

**Table 1.** Respondents' demographic characteristics and COVID-19 experience (*n* = 502)

Characteristic	Unweighted no. (weighted %)
Age group	
18–34 years old	159 (29.4)
35–49 years old	136 (23.5)
50–64 years old	123 (25.4)
≥65 years old	84 (21.8)
Education	
High school or less	162 (38.7)
Some college	75 (15.2)
College	153 (28.0)
Post college	112 (18.1)
Gender	
Female	251 (51.7)
Male	251 (48.3)
Annual income	
Less than $35k	158 (35.5)
$35k–<$75k	166 (34.6)
$75k–$100k	54 (10.3)
More than $100k	124 (19.6)
Race/ethnicity	
Non-Hispanic White	361 (62.8)
Hispanic, any race	55 (16.7)
Non-Hispanic Black	52 (12.0)
Multiple races or other race	34 (8.6)
Native Americans/Alaska Natives	2 (0.6)
Asian	23 (5.9)
Other race, including persons who identified with more than one race	9 (2.1)
Community	
Rural	99 (20.7)
Suburban	226 (46.8)
Urban	177 (32.5)
Census region	
Midwest	102 (20.7)
Northeast	93 (17.3)
South	187 (38.0)
West	120 (24.0)
COVID-19 experience	
Ill-self^a^	41 (7.9)
Ill-others^b^	119 (24.1)
Other experience^c^	342 (68.0)

a Ill-self includes been hospitalised for COVID-19; tested positive for COVID-19.

b Ill-others includes had a relative or close friend get really sick from COVID-19; had a relative or friend pass away from COVID-19.

c Other experience includes been tested for COVID-19; been in close contact (e.g. within 6 feet for 15 min total in a 24 h period) with someone who has COVID-19; had any symptom(s) of COVID-19 such as fever/chills, cough, shortness of breath, fatigue, headache, etc.; had a relative or close friend test positive for COVID-19; none of the above.

To characterise and assess factors related to holiday meal gatherings in the context of the COVID-19 pandemic, we asked respondents five questions about their experiences with COVID-19 (e.g. if ever been tested for COVID-19, been hospitalised for COVID-19), holiday gathering type (with or without NHHMs), factors influencing decisions to attend gatherings, behaviours during gatherings and prevention strategies taken before gatherings (Supplemental Material S1). Given constraints of the survey mechanism, we did not pretest to assess respondents' comprehension of questions, nor assess for interpretation (e.g. how respondents interpreted ‘good circulation and air flow’ in Question 4 of Supplementary Table S1).

### Measures

We categorised respondents' experiences with COVID-19 into three tiered groups in the following order: ill-self, ill-others and other experience (Table 2). Respondents categorised as ill-self had personal COVID-19 experiences, which included being hospitalised or testing positive for COVID-19. Respondents categorised as ill-others did not personally experience COVID-19 but reported having a relative or close friend with a severe COVID-19 experience (either hospitalised or died). Respondents categorised as other experience were respondents who did not meet the criteria for the aforementioned two categories. These categories were not mutually exclusive (e.g. a respondent in the ill-self group could have also had a relative or close friend that had a severe COVID-19 experience). Ill-self and ill-others were categorised separately because persons who had COVID-19 and recovered may be less susceptible to reinfection [13], whereas persons who had a relative or close friend with a severe COVID-19 experience but did not experience COVID-19 infection themselves remain susceptible, which might affect engagement in protective strategies. To further explore these concepts, we also analysed COVID-19 experience as two categories by combining ill-self and ill-others, and compared with other experience.

**Table 2.** Categorisation of COVID-19 experience variable

COVID-19 experience	Questionnaire response items	Category
Personal experience^a^	– Been hospitalised for COVID-19 – Tested positive for COVID-19	Ill-self
No direct personal experience, but experience through severe illness or death of relative/close friend	– Had a relative or close friend get really sick – Had a relative or friend pass away from COVID-19	Ill-other
Neither personal experience nor experience through severe illness or death of relative/close friend	– Been tested for COVID-19 – Been in close contact (e.g. within 6 feet for 15 min total in a 24 h period) with someone who has COVID-19 – Had any symptom(s) of COVID-19 such as fever/chills, cough, shortness of breath, fatigue, headache, etc. – Had a relative or close friend test positive for COVID-19 – None of the above	Other experience

a If respondents met ‘Personal experience’ criteria, they were not included in other groups.

We created variables to describe holiday gatherings with NHHMs (any meals with NHHMs *vs.* only meals with people I live with, or none of these), the meal setting (any meals indoors *vs.* only meals outdoors) and gathering size (any gatherings with >10 people *vs.* only gatherings with ≤10 people).

We asked respondents about the degree to which they considered certain factors important when they decided whether to attend holiday gatherings with NHHMs, using a five-point Likert scale of not at all important to extremely important. These were further categorised as two mutually exclusive categories: important (reported as slightly, moderately, very or extremely) *vs.* not important (reported as not at all important). Engagement in key prevention strategies and other practices before and during holiday gatherings were also measured using a five-point Likert scale and were categorised as two mutually exclusive categories: ever (reported as rarely, sometimes, often or all the time) *vs.* never. To capture respondents who always engaged in these prevention strategies during one or more holiday gatherings, we created additional mutually exclusive categories: always (all the time) *vs.* not always (never, rarely, sometimes or often). People who do not always engage in prevention strategies increase their risk of exposure to SARS-CoV-2; the less someone engages in prevention strategies, the higher the risk for exposure to SARS-CoV-2. Race/ethnicity was divided into the following mutually exclusive categories: non-Hispanic White persons, non-Hispanic Black persons, Hispanic persons (any race) or non-Hispanic multiple race/other race (including persons who were Asian, Native American/Alaska Natives, other races and persons who identified with more than one race). Because persons belonging to racial/ethnic minority groups are overrepresented among essential workers and thus may have been exposed to COVID-19 [14,15], we created an additional category that combines persons of racial/ethnic minority groups, to compare them with non-Hispanic White persons.

### Analysis

We calculated frequencies for demographic characteristics (i.e. age, gender, race/ethnicity, annual household income, education, community type, census region), COVID-19 experience, characteristics of holiday gatherings with NHHMs, key prevention strategies and other practices, and decision factors. We present frequency data as unweighted sample sizes and weighted percentages. We calculated weighted mean scores for each decision factor.

To assess the extent to which demographic characteristics, COVID-19 experience and decision factors were associated with choosing to attend holiday gatherings with NHHMs, we created a reduced variable model using a weighted main-effects multiple logistic regression with a backwards selection method utilizing Schwarz Bayesian information criteria. Demographic characteristics, listed above, and COVID-19 experience were used as statistical controls. Weighted mean scores were modelled for the decision factors. We used Zou's modified Poisson regression approach to estimate adjusted prevalence ratios (aPRs), and Tukey–Kramer Minimum Significant Difference (MSD) test to adjust confidence intervals (CI) and *P*-values to correct for familywise-error rate in cases in which all orthogonal pairings were conducted.

Using weighted main-effects multiple logistic regression and Zou's approach, we created models to assess the extent to which demographic characteristics and COVID-19 experience were associated with three key prevention strategies: wearing masks, physical distancing and avoiding crowds/gatherings. These three key strategies were selected since they have been emphasised throughout the COVID-19 pandemic, regardless of setting [16]. For this survey, this included two key prevention strategies that respondents could have engaged in during holiday gatherings (wearing masks and physical distancing (stayed at least 6 feet away from NHHMs)) and three key prevention strategies before holiday gatherings (wearing masks, physical distancing and avoiding crowds/gatherings with NHHMs). Prevention strategies before holiday gatherings were assessed because low engagement of them could increase the likelihood of exposure to SARS-CoV-2 and increase the risk of transmission during holiday gatherings. The outcomes modelled were not always engaging in these strategies.

Given the exploratory nature of the analyses, relationships at *P*⩽0.08 are discussed. All analyses were conducted using SAS version 9.4 (SAS Institute, Inc., Cary, NC, USA).

## Results

### Demographic characteristics and COVID-19 experience

Among 502 respondents, 52% were female; 63% were non-Hispanic White, 17% were Hispanic (any race), 12% were non-Hispanic Black and 9% were persons identifying with multiple or other races (6% Asian, 0.6% Native American/Alaska Natives and 2% persons who identified as another race or with more than one race) (Table 1). The median age of respondents was 43 years (interquartile range (IQR) 31–60). For COVID-19 experience, 8% of respondents were categorised as ill-self; 24% as ill-others; and 68% as other experience.

### Deciding whether to attend holiday gatherings with NHHMs

The decision factor with the highest mean score was whether an NHHM or someone at the holiday meal was at risk for severe COVID-19 illness (3.7), and the lowest mean score was pressure from family or friends to attend gatherings (2.5) (Table 3). Most respondents reported that the size of the holiday gathering was important in their decision about whether to attend (82%); more than three-quarters of respondents indicated that agreement among attendees about wearing masks at all times when not eating or drinking was important in their decision about whether to attend (78%).

**Table 3.** Decision factors and their importance in determining to attend holiday gatherings with non-household members

How important were the following factors in your decision about whether to have holiday meals with people you do not live with?^a^	Important, no. (weighted %)^b^^,^^c^	Not important, no. (weighted %)^c^^,^^d^	Mean	Standard deviation
Whether someone I live with or someone at the holiday meal is at risk for severe COVID-19 illness	455 (90.5)	47 (9.7)	3.68	1.32
Whether other meal guests typically practice social distancing and wear masks before attending gathering	435 (86.5)	67 (13.5)	3.55	1.40
Whether other meal guests were a part of my ‘COVID bubble’ (i.e. people who I do not live with but feel safe to be around)	437 (86.2)	65 (13.8)	3.52	1.40
The number of COVID-19 cases in the community where your holiday meal would occur or the community where guests were coming from	440 (87.9)	62 (12.1)	3.47	1.36
Centers for Disease Control and Prevention's (CDC's) holiday guidance	436 (86.7)	66 (13.3)	3.46	1.37
The number of guests from other households attending	419 (82.3)	83 (17.7)	3.41	1.46
Recommendations from local or state government	433 (86.2)	69 (13.8)	3.40	1.38
Social distancing that would take place during the meal (e.g. spaced seating)	416 (82.7)	86 (17.4)	3.39	1.45
Desire to see friends and family	444 (88.0)	58 (12.1)	3.39	1.31
The location where the holiday meal would be served (outside or inside)	410 (81.3)	92 (18.8)	3.28	1.44
Agreement that everyone would wear masks at all times while not eating or drinking	391 (77.5)	111 (22.4)	3.23	1.53
Pressure from my family or friends to attend gatherings	316 (61.1)	186 (38.9)	2.49	1.47

a Five-point Likert scale was used including not at all important, slightly, moderately, very or extremely important.

b Respondent reported slightly, moderately, very or extremely important.

c Responses were weighted by gender, age, community type, census region, race/ethnicity, household income, employment status and education to reflect US Current Population Survey proportions.

d Respondent reported not at all important.

### Predictors associated with attending holiday gatherings with NHHMs

About one-third of respondents attended at least one holiday gathering with NHHMs (34%). Several decision factors, age and COVID-19 experience were associated with attending holiday gatherings with NHHMs (Table 4). Respondents were more likely to attend NHHM gatherings if they reported desire to see friends and family (aPR = 1.26, 95% CI 1.13–1.40) and whether other guests were part of their ‘COVID-19 bubble’ (aPR 1.30, 95% CI 1.18–1.44) as more important in their decision to attend. Respondents were more likely to attend NHHM gatherings if they were younger (18–34 years) compared with respondents who were 35 or older (aPR 1.44, 95% CI 1.12–1.86), or if they had a relative or close friend experience severe COVID-19 compared with respondents with other COVID-19 experience (aPR 1.37, 95% CI 1.00–1.87), though this was marginally significant. Respondents were *less* likely to attend NHHM gatherings if they reported CDC's holiday guidance as more important (aPR 0.82, 95% CI 0.72–0.92) and agreements that everyone would wear masks at all times while not eating or drinking (aPR 0.81, 95% CI 0.73–0.89) as more important in their decision to attend.

**Table 4.** Predictors associated with attending a holiday gathering with non-household members^a^ (*n* = 502)

Comparison	Adjusted prevalence ratio (adjusted 95% CI^b^)	Adjusted *P*-values^b^
Age group (overall *P*-value *=* 0.051)		
18–34 *vs*. 35–49 years old	1.37 (0.91–2.07)	0.193
18–34 *vs.* 50–64 years old	1.54 (0.98–2.40)	0.064
18–34 *vs.* ≥65 years old	1.42 (0.88–2.31)	0.244
35–49 *vs.* 50–64 years old	1.12 (0.68–1.84)	0.937
35–49 *vs.* ≥65 years old	1.04 (0.62–1.74)	0.998
50–64 *vs.* ≥65 years old	0.93 (0.54–1.59)	0983
18–34 *vs.* ≥35 years old	**1.44** (**1.12**–**1.86)**	**0.005**^c^
Sex (overall *P*-value *=* 0.075)		
Male *vs.* female	1.25 (0.98–1.60)	0.071
COVID-19 experience (overall *P*-value *=* 0.077)		
Ill-others^d^ *vs.* ill-self^e^	1.07 (0.61–1.89)	0.958
Ill-self^e^ *vs.* other experience^f^	1.28 (0.74–2.19)	0.541
Ill-others^d^ *vs.* other experience^f^	1.37 (1.00–1.87)	0.054
Ill-self^e^ or ill-others^d^ *vs.* other experience^f^	1.32 (0.99–1.75)	0.087‡
Decision factors		
Desire to see friends and family	**1.26** (**1.13**–**1.40)**	**<0**.**001**
Whether other meal guests were a part of my ‘COVID bubble’ (i.e. people who I do not live with but feel safe to be around)	**1.30** (**1.18**–**1.44)**	**<0**.**001**
Agreement that everyone would wear masks at all times while not eating or drinking	**0.81** (**0.73**–**0.89)**	**<0**.**001**
Centers for Disease Control and Prevention's (CDC's) holiday guidance	**0.82** (**0.72**–**0.92)**	**0**.**001**

*Note*: Results significant at *P*  0.05 are bolded.

a Reduced weighted multiple logistic regression model. Characteristics including age, gender, race/ethnicity, annual household income, education, community type, census region and COVID-19 experience were used as statistical controls.

b Tukey–Kramer MSD used to adjust for multiple comparisons.

c Planned comparison. Tukey–Kramer MSD adjustment not applied.

d Ill-others includes had a relative or close friend get really sick from COVID-19; had a relative or friend pass away from COVID-19.

e Ill-self includes been hospitalised for COVID-19; tested positive for COVID-19.

f Other experience includes been tested for COVID-19; been in close contact (e.g. within 6 feet for 15 min total in a 24 h period) with someone who has COVID-19; had any symptom(s) of COVID-19 such as fever/chills, cough, shortness of breath, fatigue, headache, etc.; had a relative or close friend test positive for COVID-19; none of the above.

### Key prevention strategies and other practices during holiday gatherings with NHHMs

Among the respondents who did have holiday gatherings with NHHMs, most only attended gathering(s) with ≤10 people (81%). Three-quarters of respondents had at least one gathering indoors (76%). Other practices included eating outdoors (ever: 52%, always: 7%), eating outdoors in a tent or enclosure (ever: 42%, always: 5%), eating indoors without good circulation (ever: 61%, always: 6%), sitting at a table only with household members with tables spaced at least 6 feet apart (ever: 72%, always: 16%), and bringing their own food/drinks (ever: 71%, always: 11%).

During holiday gatherings with NHHMs, key prevention strategies that respondents ever or always engaged in included wearing a mask except when eating or drinking (ever: 73%, always: 29%) and staying at least 6 feet away from NHHMs (ever: 84%, always: 23%) (Table 5). Only one of the two key prevention strategies during holiday gatherings was significantly associated with any predictors (Table 6). Respondents who had other experience with COVID-19 were more likely to not always wear masks compared with respondents in the combined ill-self/ill-others category (aPR 1.38, 95% CI 1.02–1.88).

**Table 5.** Key prevention strategies and other practices during holiday gatherings with non-household members (*n* = 176)

Description	Ever^a^, no. (weighted %)^b^	Never, no. (weighted %)^b^	Always, no. (weighted %)^b^	Not always^c^, no. (weighted %)^b^
Wearing a mask except when eating or drinking^d^	131 (72.7)	45 (27.4)	50 (28.8)	126 (71.3)
Staying at least 6 feet away from people who I do not live with^d^	148 (83.9)	28 (16.2)	40 (23.4)	136 (76.7)
Sitting at a table only with people who I live with, with tables spaced at least 6 feet apart from others	128 (72.1)	48 (28.7)	27 (16.0)	149 (84.0)
Bringing my own food or drinks	127 (71.3)	49 (28.7)	18 (10.9)	158 (89.1)
Eating outdoors (not inside a tent or any type of enclosure)	95 (51.6)	81 (48.4)	10 (6.7)	166 (93.3)
Eating outdoors in a tent or enclosure	81 (41.8)	95 (58.1)	8 (5.1)	168 (94.8)
Eating indoors in a location that did not appear to have good circulation and air flow	111 (60.5)	65 (39.5)	13 (6.0)	163 (94.0)

a Ever is reported as rarely, sometimes, often or all the time.

b Responses were weighted by gender, age, community type, census region, race/ethnicity, household income, employment status and education to reflect US Current Population Survey proportions.

c Not always is reported as never, rarely, sometimes or often.

d This is a Centers for Disease Control and Prevention (CDC) key prevention strategy.

**Table 6.** Predictors associated with key prevention strategies during holiday gatherings with non-household members (*n* = 176)

Prevention strategy	Comparison	Adjusted prevalence ratio (adjusted 95% CI^a^)	Adjusted *P*-values^a^
*Not always* wearing a mask (except when eating or drinking)	COVID-19 experience (overall *P*-value = 0.057)		
	Other experience^b^ *vs*. ill-self^c^	1.54 (0.80–2.99)	0.272
	Other experience^b^ *vs.* ill-others^d^	1.24 (0.94–1.63)	0.171
	Ill-others^d^ *vs.* ill-self^c^	1.25 (0.62–2.51)	0.736
	Other experience^b^ *vs.* ill-self^c^ or ill-others^d^	**1.38** (**1.02**–**1.88)**	**0.039**^e^

*Note*: Results significant at *P *⩽** 0.05 are bolded.

a Tukey–Kramer MSD used to adjust for multiple comparisons. Characteristics including age, gender, race/ethnicity, annual household income, education, community type, census region and COVID-19 experience were used as statistical controls.

b Other experience includes been tested for COVID-19; been in close contact (e.g. within 6 feet for 15 min total in a 24 h period) with someone who has COVID-19; had any symptom(s) of COVID-19 such as fever/chills, cough, shortness of breath, fatigue, headache, etc.; had a relative or close friend test positive for COVID-19; none of the above.

c Ill-self includes been hospitalised for COVID-19; tested positive for COVID-19.

d Ill-others includes had a relative or close friend get really sick from COVID-19; had a relative or friend pass away from COVID-19.

e Planned comparison. Tukey–Kramer MSD adjustment not applied.

### Key prevention strategies and other practices before holiday gatherings with NHHMs

During the 2 weeks before attending NHHM holiday gatherings, key prevention strategies that respondents ever or always engaged in included wearing a mask every time they were around NHHMs (ever: 91%, always: 39%), staying at least 6 feet away from NHHMs (ever: 94%, always: 30%) and avoiding group gatherings with NHHMs (ever: 91%, always: 29%) (Table 7). Other practices included avoiding indoor public places (ever: 93%, always: 37%), avoiding grocery stores (ever: 64%, always: 9%), quarantining for 14 days (ever: 55%, always: 14%) and getting tested for COVID-19 at least 3–5 days before holiday gatherings with NHHMs (ever: 43%, always: 9%). Several factors were significantly associated with not always engaging in two of the three key prevention strategies before attending NHHM holiday gatherings (Table 8). Younger adults (18–34 years) were more likely to not always wear masks around NHHMs during the 2 weeks before holiday gatherings than respondents aged ≥35 years (aPR 1.53, 95% CI 1.19–1.97). Respondents with other COVID-19 experience were more likely to not always wear masks before gatherings than respondents in the combined ill-self/ill-others category (aPR 1.56, 95% CI 1.18–2.07). Although none of the individual racial/ethnic pairwise comparisons were significant, respondents who identified as non-Hispanic White were more likely to not always wear masks before gatherings than respondents who identified with any racial/ethnic minority group (aPR 1.57, 95% CI 1.13–2.18). Respondents with other COVID-19 experience were more likely to not always avoid group gatherings with NHHMs during the 2 weeks before holiday gatherings as compared with respondents in the combined ill-self/ill-others category (aPR 1.36, 95% CI 1.06–1.76).

**Table 7.** Key prevention strategies and other practices during the 2 weeks before holiday gatherings with non-household members (*n* = 176)

Description	Ever^a^, no. (weighted %)^b^	Never, no. (weighted %)^b^	Always, no. (weighted %)^b^	Not always^c^, no. (weighted %)^b^
Wore a mask every time I was around people I do not live with^d^	157 (91.0)	19 (9.0)	64 (39.0)	112 (61.0)
Staying at least 6 feet away when I was around people I do not live with^d^	163 (94.1)	13 (6.0)	50 (30.4)	126 (69.6)
Avoiding group gatherings with people I do not live with^d^	159 (90.8)	17 (9.2)	49 (29.2)	127 (70.8)
Avoiding indoor public places (including gyms, restaurants, bars and stores (other than grocery stores))	164 (92.8)	12 (7.2)	59 (36.5)	117 (63.5)
Avoiding going to grocery stores and had food delivered instead	114 (64.2)	62 (35.8)	17 (8.7)	159 (91.3)
Quarantining for 14 days prior to having holiday meals with people I do not live with	100 (55.4)	76 (44.6)	23 (13.6)	153 (86.4)
Getting tested for COVID-19 at least 3–5 days prior to having holiday meals with people I do not live with	81 (43.4)	25 (13.4)	16 (9.4)	160 (90.6)

a Ever is reported as rarely, sometimes, often or all the time.

b Responses were weighted by gender, age, community type, census region, race/ethnicity, household income, employment status and education to reflect US Current Population Survey proportions.

c Not always is reported as never, rarely, sometimes or often.

d This is a Centers for Disease Control and Prevention (CDC) key prevention strategy.

**Table 8.** Predictors associated with key prevention strategies during the 2 weeks before holiday gatherings with non-household members (*n* = 176)

Prevention strategy	Comparison	Adjusted prevalence ratio (adjusted 95% CI^a^)	Adjusted *P*-values^a^
*Not always* wearing a mask every time I was around people I do not live with	Age group (overall *P*-value = 0.011)		
	18–34 *vs.* 35–49 years old	1.38 (0.94–2.01)	0.135
	18–34 *vs.* 50–64 years old	1.50 (0.98–2.30)	0.066
	18–34 *vs.* ≥65 years old	1.72 (0.97–3.05)	0.071
	35–49 *vs.* 50–64 years old	1.09 (0.68–1.76)	0.963
	35–49 *vs.* ≥65 years old	1.25 (0.68–2.31)	0.781
	50–64 *vs.* ≥65 years old	1.14 (0.62–2.10)	0.941
	18–34 *vs.* ≥35 years old	**1.53** (**1.19**–**1.97)**	**0.001**^b^
	Race/ethnicity^c^ (overall *P*-value = 0.047)		
	Non-Hispanic White *vs.* Hispanic persons	1.26 (0.80–1.99)	0.544
	Non-Hispanic White persons *vs.* people of multiple races or other race	1.63 (0.67–3.95)	0.484
	Non-Hispanic White *vs.* Non-Hispanic Black persons	1.87 (0.86–4.08)	0.161
	Hispanic persons *vs.* people of multiple races or of other race	1.29 (0.49–3.39)	0.905
	Hispanic *vs.* Non-Hispanic Black persons	1.48 (0.61–3.58)	0.661
	People of multiple races or other race *vs.* Non-Hispanic Black persons	1.15 (0.35–3.77)	0.991
	Non-Hispanic White persons *vs.* people of racial/ethnic minority groups^d^	**1.57** (**1.13**–**2.18)**	**0.007**^b^
	Census region (overall *P*-value = 0.024)		
	South *vs.* West	1.08 (0.75–1.54)	0.951
	South *vs.* Northeast	1.42 (0.88–2.29)	0.231
	South *vs.* Midwest	**1.68** (**0.99–2.86)**	**0**.**057**
	West *vs.* Northeast	1.32 (0.82–2.13)	0.449
	West *vs.* Midwest	1.56 (0.90–2.70)	0.155
	Northeast *vs.* Midwest	1.18 (0.63–2.22)	0.901
	COVID-19 experience (overall *P*-value = 0.004)		
	Other experience^e^ *vs.* ill-self^f^	1.56 (0.94–2.60)	0.100
	Other experience^e^ *vs.* ill-others^g^	**1.56** (**1.04**–**2.34)**	**0**.**027**
	Ill-others^g^ *vs.* ill-self^f^	1.00 (0.53–1.88)	0.999
	Other experience^e^ *vs.* ill-self^f^ or ill-others^g^	**1.56** (**1.18**–**2.07)**	**0.002**^b^
*Not always* avoiding group gatherings with people I do not live with	COVID-19 experience (overall *P*-value = 0.032)		
	Other experience^e^ *vs.* ill-self^f^	1.45 (0.86–2.45)	0.217
	Other experience^e^ *vs.* ill-others^g^	1.28 (0.95–1.72)	0.122
	Ill-others^g^ *vs.* ill-self^f^	1.13 (0.63–2.04)	0.873
	Other experience^e^ *vs.* ill-self^f^ or ill-others^g^	**1.36** (**1.06**–**1.76)**	**0.018**^b^

*Note*: Results significant at *P *⩽** 0.05 are bolded.

a Tukey–Kramer MSD used to adjust for multiple comparisons. Characteristics including age, gender, race/ethnicity, annual household income, education, community type, census region and COVID-19 experience were used as statistical controls.

b Planned comparison. Tukey–Kramer MSD adjustment not applied.

c Race/ethnicity was divided into the following mutually exclusive categories: non-Hispanic White, non-Hispanic Black, Hispanic (any race), multiple races or other race (Asian, Native American/Alaska Natives, other and persons who identify with more than one race).

d Includes all races/ethnicities other than non-Hispanic White.

e Other experience includes been tested for COVID-19; been in close contact (e.g. within 6 feet for 15 min total in a 24 h period) with someone who has COVID-19; had any symptom(s) of COVID-19 such as fever/chills, cough, shortness of breath, fatigue, headache, etc.; had a relative or close friend test positive for COVID-19; none of the above.

f Ill-self includes been hospitalised for COVID-19; tested positive for COVID-19.

g Ill-others includes had a relative or close friend get really sick from COVID-19; had a relative or friend pass away from COVID-19.

## Discussion

Most respondents followed CDC's COVID-19 holiday gathering guidance either by gathering only with people who lived in their household (66%) or by engaging in key prevention strategies to make gatherings with people outside their household safer, like wearing masks (73%) and practicing physical distancing (84%). Engagement in these same prevention strategies during the 2 weeks before holiday gatherings was even higher; 91% wore masks, and 94% practiced physical distancing around NHHMs. Furthermore, the more important that (1) CDC guidance and (2) agreement among guests to wear masks when not eating or drinking was in a respondent's decision whether to attend holiday gatherings, the less likely respondents were to actually attend them. These findings suggest that CDC's COVID-19 guidance may be reaching the public and underscores the importance of continuing to share public health messaging during the COVID-19 pandemic.

Although many respondents who attended gatherings engaged in key prevention strategies before and during holiday gatherings with people outside their household, most respondents did not always engage in these prevention strategies, which increases the risk of SARS-CoV-2 transmission. During holiday gatherings, less than one-third of respondents always wore masks when not eating or drinking (29%) or always practiced physical distancing (23%). Similarly, before holiday gatherings, fewer respondents always wore masks when not eating or drinking (39%) and practiced physical distancing (30%). Consistent and correct mask usage is critical for reducing SARS-CoV-2 transmission among those not fully vaccinated [17,18], particularly when gathering indoors with people outside of one's household [19–23]. When mask use is challenging, like when eating and drinking, good ventilation and physical distancing are especially important.

Younger respondents were more likely to attend holiday gatherings and were more likely to not always wear masks during the 2 weeks before them. This finding is consistent with previous studies which identified reduced mask adherence among young adults [24] and social, peer pressure and low perceived severity of COVID-19 outcomes as possible drivers of behaviour [25,26]. Though younger respondents were more likely to not always wear masks *before* gatherings, there was no association between age and mask usage *during* holiday gatherings. One study found that even though young adults perceived COVID-19 severity for themselves as low, perceived severity for others was high, which might explain why young adults might not wear masks with peers, but would wear masks when with loved ones at increased risk for severe illness [26], including at holiday gatherings.

Respondents who identified as non-Hispanic White were more likely to not always wear masks before holiday gatherings than persons of racial/ethnic minority groups. This finding is consistent with a previous study which identified reduced mask adherence among White persons compared with Latina/o, Black and Asian persons, though ethnicity was not captured [27]. Higher mask adherence among racial/ethnic minority groups may be explained by a heightened awareness of the overrepresentation of Hispanic, non-Hispanic Black and non-Hispanic American Indian/Alaska Native persons in COVID-19 incidence, emergency department visits, hospitalisations and deaths compared with non-Hispanic White persons [28–31]. Moreover, mask adherence could be related to the perceptions of susceptibility to COVID-19 – a known predictor of behaviour across diseases [32]. Though, a recent study found lower perceived susceptibility of COVID-19 among Black persons compared to White persons – data consistent with perceptions during H5N1 and HINI outbreaks [33–35]. More research is needed to understand why persons choose to wear a mask during COVID-19, and the relationship between perceived susceptibility of COVID-19 and mask adherence, especially among racial/ethnic groups that have been disproportionately affected by COVID-19.

Respondents who had not experienced COVID-19 themselves or had a relative or close friend experience severe COVID-19 were more likely to not always wear a mask during or before holiday gatherings and were more likely to not always avoid group gatherings. A lack of COVID-19 experience may contribute to reduced perceptions of susceptibility to infection and severity of disease. In one study, knowing someone with COVID-19 was a predictor of mask wearing [36]. In another, persons who experienced COVID-19 themselves had an increased perception of severity and described a sense of fear that they would infect others [37]. Perceived severity and susceptibility are two constructs that are known to be associated with human behaviour and have been identified as predictors of COVID-19 mitigation behaviours in other studies [25,36,38]. In contrast, respondents who had a relative or close friend who had experienced severe COVID-19 were more likely to attend holiday gatherings with others outside their household than those with other experience. This may be driven by multiple factors including COVID-19 pandemic fatigue causing relaxation of prevention behaviours [36,39], or that the desire to continue traditional holiday rituals superseded perceived risk. We found that the more important the desire to see friends or family was in the decision to attend a holiday gathering, the more likely a respondent was to attend, suggesting this may be an important driver. Of note, we did not assess the timing of respondent's relative or close friend's severe COVID-19 experience, or whether respondents attended holiday gatherings with the same relatives/close friends who had a severe COVID-19 experience.

While we hypothesised that associations between attending holiday gatherings or engagement in key prevention strategies and COVID-19 experience might differ between ill-self and ill-others categories, we did not find strong evidence for this. For each model, the ill-self and ill-others categories were not significantly different from one another, and they each had adjusted prevalence ratios that were similar in magnitude and direction when compared with the other COVID-19 experience category. Our inability to detect differences between these groups may be partly due to small sample size; only 8% of respondents in the overall survey were categorised as ill-self. Another explanation could be how we categorised COVID-19 experience. Respondents categorised as ill-self could have also had a relative/close friend experience severe COVID-19, since ill-self and ill-others were not mutually exclusive. Further, respondents categorised as other COVID-19 experience included people who experienced COVID-19-like symptoms; these respondents may have perceived they had COVID-19, even if they did not receive a positive test result. One study found a positive association between persons who had COVID-19-like symptoms and wearing masks [36]. By assessing COVID-19 illness based on test results, rather than one's perception of having COVID-19, we may have underestimated their perceived susceptibility to subsequent COVID-19 illness, which in turn could have influenced their behaviours.

Attending future holiday gatherings of any size with people outside one's household poses some degree of risk of SARS-CoV-2 transmission, particularly if indoors, and if attendees have not been fully vaccinated against COVID-19. The more a person who is not fully vaccinated interacts with other people who are not fully vaccinated without engaging in prevention strategies (e.g. indoors and without masks), both during and before gatherings, and the longer that interaction lasts, the higher the risk of becoming infected with, or spreading, SARS-CoV-2 [40]. Given the high proportion of respondents who reported eating indoors (which could be due to factors such as cold weather) in this survey, promoting outdoor dining during warmer months could be an effective strategy for reducing transmission. Messaging should also continue to emphasise key prevention strategies for people who are not fully vaccinated during and before gatherings. Messaging around mask usage in particular could focus on younger adults who are not fully vaccinated, non-Hispanic White persons, and persons who have not experienced COVID-19 personally or had a relative/close friend experience severe COVID-19. Messaging to leverage others' experiences with COVID-19 could include storytelling or developing public health narratives, such as CDCs ‘I wear a mask because’ campaign [41]. These narratives can be helpful in addressing reduced perceptions of susceptibility and severity, overcoming resistance to public health messaging, and supporting observational learning, and they may positively influence certain subpopulations who might identify with the narrative [42]. Communication strategies could also consider ways to strengthen consistency of engagement in these prevention strategies.

### Limitations

This study is subject to at least six limitations. First, though quota sampling and weighting survey data were employed to increase representativeness, data collected may not be generalisable across the US population given non-probability sampling and poor response rate (31.5%). Second, self-reported data may be impacted by social desirability, recall, volunteer, or recency bias. Third, although we attempted to capture driving factors behind decisions around attending holiday gatherings, this exploratory study was not designed to understand reasoning behind certain behaviours. Fourth, small sample sizes limited statistical power and thus our ability to conduct sub-analyses. Fifth, we did not capture certain holiday gathering characteristics, including if NHHMs attending holiday gatherings were only part of one's COVID-19 bubble, or the total number of holiday gatherings that respondents attended. Sixth, timing of COVID-19 experience relative to holiday gatherings was not assessed; therefore, we are unable to determine if COVID-19 experience directly impacted decisions and engagement in prevention strategies during and before holiday gatherings. This analysis was not designed to determine whether engagement in prevention strategies differed between gatherings for respondents who may have attended multiple gatherings.

## Conclusion

Most respondents heeded CDC's 2020 fall/winter holiday guidance and did not attend holiday gatherings with NHHM; among respondents who did, many engaged in a range of prevention strategies and other practices to make them safer, although not consistently. Despite these efforts, improvements in the consistency of practicing key prevention strategies is needed and could further help slow the spread of COVID-19. Future research could focus on characterizing factors associated with practicing prevention strategies periodically *vs.* consistently. Future vaccination efforts are particularly critical as a prevention strategy for those attending indoor holiday gatherings since many respondents did not consistently engage in key prevention strategies.

## Data Availability

If interested in accessing data or other materials related to this manuscript, readers may contact the corresponding author.

## Footnotes

1 45 C.F.R. part 46, 21 C.F.R. part 56; 42 U.S.C. Sect. 241(d); 5 U.S.C. Sect. 552a; 44 U.S.C. Sect. 3501 *et seq*.

## Supplementary material

For supplementary material accompanying this paper visit https://doi.org/10.1017/S0950268822000115. click here to view supplementary material
