# Supplementary material for: Decisions to attend holiday gatherings during COVID-19 and engagement in key prevention strategies: United States, January 2021
Source: Epidemiol Infect. 2022 Feb 9;150:e32. doi: 10.1017/S0950268822000115 (PMC8886078; doi:10.1017/S0950268822000115)
Supplement: Supplementary file 1 [file S0950268822000115sup001.docx]

**Supplementary Table S1. Holiday Meal Survey Questions**

1. Which of the following has ever happened to you? [Select as many as apply for 01-08]

01 Been in close contact (e.g., within 6 feet for 15 minutes total in a 24-hour period) with someone who has COVID-19

02 Been tested for COVID-19

03 Tested positive for COVID-19

04 Had any symptom(s) of COVID-19 such as fever/chills, cough, shortness of breath, fatigue, headache, etc.

05 Been hospitalized for COVID-19

06 Had a relative or close friend test positive for COVID-19

07 Had a relative or close friend get really sick from COVID-19

08 Had a relative or friend pass away from COVID-19

09 None of the above [EXCLUSIVE]

2. Which of the following describe the types of holiday meal gatherings you had during the 2020 holiday season (for example, Thanksgiving, Hanukah, Christmas, New Year’s)? [Select as many as apply for 01-05]

01 I had holiday meals only with people I live with

02 I had holiday meals outdoors with 10 or fewer people I do not live with

03 I had holiday meals indoors with 10 or fewer people I do not live with

04 I had holiday meals outdoors with more than 10 people I do not live with

05 I had holiday meals indoors with more than 10 people I do not live with

06 None of these [EXCLUSIVE]

3. How important were the following factors in your decision about whether to have holiday meals with people you do not live with? [Select one answer for each.]

01 Not at all important

02 Slightly important

03 Moderately important

04 Very important

05 Extremely important

A. The number of COVID-19 cases in the community where your holiday meal would occur or the community where guests were coming from

B. Desire to see friends and family

C. Pressure from my family or friends to attend gatherings

D. Whether other meal guests typically practice social distancing and wear masks before attending gathering

E. Whether other meal guests were a part of my ‘COVID-19 bubble’ (i.e., people who I do not live with but feel safe to be around)

F. The location where the holiday meal would be served (outside or inside)

G. Social distancing that would take place during the meal (e.g., spaced seating)

H. Agreement that everyone would wear masks at all times while not eating or drinking

I. The number of guests from other households attending

J. Whether someone I live with or someone at the holiday meal is at risk for severe COVID-19 illness

K. Recommendations from local or state government

L. Centers for Disease Control and Prevention’s (CDC’s) holiday guidance

4. (If selected 2-5 for Question 2) During the holiday meals you had with people you do not live with, how often did you engage in the following activities because of COVID-19? [Select one answer for each; [ASK IF #2 (02-05)]

01 Never

02 Rarely

03 Sometimes

04 Often

05 All the time

A. I ate indoors in a location that appeared to have good circulation and air flow

B. I ate indoors in a location that did not appear to have good circulation and air flow

C. I ate outdoors (not inside a tent or any type of enclosure)

D. I ate outdoors in a tent or enclosure

E. I wore a mask except when eating or drinking

F. I stayed at least 6 feet away from other people who I do not live with

G. I sat at a table only with people who I live with, with tables spaced at least 6 feet apart from others

H. I brought my own food or drinks

5. (If selected 2-5 for Question 2) How often did you take the following preventive measures during the two weeks before having holiday meals with people you do not live with? [Select one answer for each.]

01 Never

02 Rarely

03 Sometimes

04 Often

05 All the time

A. Avoided indoor public places, including gyms, restaurants, bars, and stores (other than grocery stores)

B. Avoided going to grocery stores and had food delivered instead

C. Avoided group gatherings with people I do not live with

D. Avoided either going into the home of anyone I don’t live with or having anyone I don’t live with enter my home

E. Wore a mask every time I was around people I do not live with

F. Stayed at least 6 feet away when I was around people I do not live with

G. Quarantined for 14 days prior to having holiday meals with people I do not live with

H. Got tested for COVID-19 at least 3-5 days prior to having holiday meals with people I do not live with
